# Trial Design for a Diagnostic Accuracy Study of a Point-of-Care Test for the Detection of *Taenia solium* Taeniosis and (Neuro)Cysticercosis in Community Settings of Highly Endemic, Resource-Poor Areas in Zambia: Challenges and Rationale

**DOI:** 10.3390/diagnostics11071138

**Published:** 2021-06-22

**Authors:** Inge Van Damme, Chiara Trevisan, Kabemba E. Mwape, Veronika Schmidt, Pascal Magnussen, Gideon Zulu, Chishimba Mubanga, Dominik Stelzle, Emmanuel Bottieau, Emmanuel Abatih, Isaac K. Phiri, Maria V. Johansen, Chishala Chabala, Andrea S. Winkler, Pierre Dorny, Sarah Gabriël, on behalf of the SOLID Consortium

**Affiliations:** 1 Department of Veterinary Public Health and Food Safety, Faculty of Veterinary Medicine, Ghent University, 9820 Merelbeke, Belgium; ctrevisan@itg.be (C.T.); chishimbamubanga@yahoo.com (C.M.); 2 Department of Biomedical Sciences, Institute of Tropical Medicine, 2000 Antwerp, Belgium; pdorny@itg.be; 3 Department of Clinical Studies, School of Veterinary Medicine, University of Zambia, Lusaka 10101, Zambia; evans.mwape@unza.zm (K.E.M.); gideonzulu@yahoo.com (G.Z.); igphiri@yahoo.co.uk (I.K.P.); 4 Department of Neurology, Center for Global Health, Klinikum Rechts der Isar, Technical University of Munich, 81675 Munich, Germany; veronika.schmidt@tum.de (V.S.); dominik.stelzle@tum.de (D.S.); andrea.winkler@tum.de (A.S.W.); 5 Centre for Global Health, Institute of Health and Society, Faculty of Medicine, University of Oslo, 0450 Oslo, Norway; 6 Faculty of Health and Medical Sciences, University of Copenhagen, 2200 Copenhagen, Denmark; pma@sund.ku.dk; 7 Ministry of Health, Lusaka 10101, Zambia; 8 Department of Clinical Sciences, Institute of Tropical Medicine, 2000 Antwerp, Belgium; EBottieau@itg.be; 9 Department of Applied Mathematics, Computer Sciences and Statistics, Faculty of Sciences, Ghent University, 9000 Ghent, Belgium; Emmanuel.Abatih@UGent.be; 10 Independent Researcher, 2100 Copenhagen, Denmark; mariavangjohansen@gmail.com; 11 Paediatrics and Child Health, Children’s Hospital, University Teaching Hospitals, Lusaka 10101, Zambia; cchabala@gmail.com; 12 Department of Paediatrics and Child Health, School of Medicine, University of Zambia, Lusaka 10101, Zambia

**Keywords:** diagnosis, accuracy, sensitivity, specificity, *Taenia solium*, taeniosis, cysticercosis, POC test, low-resource settings, point-of-care

## Abstract

Field-applicable, high-quality, and low-cost diagnostic tools are urgently needed for *Taenia solium*. The aim of this paper is to describe the design, challenges, and rationale for the design of a diagnostic accuracy study in low-resource community settings in Zambia. The trial was designed as a prospective study with a two-stage design to evaluate a new point-of-care test (TS POC) for the detection of taeniosis and (neuro)cysticercosis. Participants within randomly selected households were tested with the TS POC test (index test). Participants who tested TS POC positive for taeniosis and/or cysticercosis and a subset of the negatives were requested to give blood and stool samples for reference testing, and to undergo clinical examination and a cerebral CT scan. The difficulties of conducting a clinical trial in settings with limited research and neuroimaging infrastructure as well as peculiarities specifically related to the disease (low prevalence of taeniosis and the lack of a gold standard) were taken into consideration for the design of this study. The two-stage design increased the efficiency of the study by reducing the number of samples, clinical examinations, and CT scans. Simplified flows and sampling processes were preferred over complex follow-up and randomization systems, aiming to reduce bias and increase the generalizability of the study.

## 1. Introduction

*Taenia solium* is a neglected zoonotic parasite ranked first on the global scale of foodborne parasites [1]. Humans are the final host, carrying the tapeworm in their intestines (taeniosis, T), but can also act as an accidental dead-end intermediate host, in which the metacestode larval stage can develop in the muscles, the subcutaneous tissue (cysticercosis, CC) or the central nervous system (neurocysticercosis, NCC). *T. solium* is endemic in sub-Saharan Africa, Latin America, and south-east Asia [2], particularly affecting poor communities with free-range pigs and low levels of sanitation, hygiene, and education. In rural communities of Zambia, high prevalence rates of *T. solium* have been reported and NCC is considered the main cause of acquired epilepsy in these areas [3,4,5]. Apart from the significant public health impact, NCC also has a strong economic and social impact on people suffering from or affected by this disease, as they are often stigmatized and marginalized in their communities.

The diagnosis of NCC in such settings is problematic, particularly because it requires access to neuroimaging and trained neurologists, which are scarce in resource-poor areas. Also, for the diagnosis of CC and T, the existing tests are not suitable to use in these communities because an accurate detection is either expensive, time consuming, and/or requires expensive equipment, infrastructure, and/or skilled personnel [6], all of which are scarce in resource-limited settings. Therefore, field applicable, affordable, and accurate point-of-care (POC) tests for T and (N)CC are urgently needed in order to control the pathogen. Such tests are not only required for diagnosis and treatment, but also for surveillance and monitoring of control programs [7]. Although several rapid diagnostic tests have been developed, many still require proper evaluation in the population in which they are intended to be used [8]. Therefore, the SOLID project “Evaluation of an antibody detecting point-of-care test for the diagnosis of *Taenia solium* taeniosis and (neuro)cysticercosis in Tanzania and Zambia” was launched in 2016. Within the project, the performance of a newly developed POC test was assessed in two different settings to represent the intended use and role where the test may be used in practice: community-based (in Zambia) and hospital-based (in Tanzania).

The conduct of a diagnostic accuracy study in low-resource community settings was anticipated to be challenging. Besides the difficulties of conducting a clinical trial in developing countries (such as the limited research and neuroimaging infrastructure mentioned above), peculiarities specifically related to the disease led to additional concerns for the design and planning of this study. The low prevalence of taeniosis and the lack of a gold standard for taeniosis and cysticercosis were important factors to consider when designing the trial. Therefore, the aim of this paper is to describe the design, the challenges, and rationale for the design of the diagnostic accuracy study in low-resource community settings in Zambia. The materials and methods section presents the TS POC test (index test under evaluation), gives an overview of the planning of the study and describes the design that was finally used for the trial (based on protocol version 1.3; 29 August 2017), which takes all the challenges into account. In the discussion, we detail the rationale behind different design aspects that were chosen for this trial, aiming to reduce bias and increase the generalizability of the study results.

## 2. Materials and Methods

### 2.1. TS POC Test

An antibody-detecting prototype test (TS POC) has been developed by a team of researchers from the Centers for Disease Control and Prevention (CDC), Atlanta, USA, in collaboration with the Department of Neurology, Klinikum rechts der Isar, Technical University of Munich (TUM), Germany. The TS POC test combines two well-validated and extensively used recombinant proteins in one multi-strip test kit: rT24H for the detection of CC antibodies (TS POC CC), and rES33 for the detection of T antibodies (TS POC T) [9,10]. Recombinant antigens were chosen for the present TS POC prototype to facilitate a future standardized low-cost production of this test for resource-poor settings. The preliminary laboratory performance of the TS POC was estimated at a sensitivity of 88–93% (for one cyst or multiple cysts, respectively) and specificity of 99% for cysticercosis, and a sensitivity of 82% and specificity of 99% for taeniosis (CDC, TUM, 2017 unpublished data). Before the potential commercialization and implementation of the test, a large-scale field evaluation of its performance and applicability as a rapid test is required. Therefore, a diagnostic accuracy study was conducted, aiming to assess the sensitivity and specificity of the multistrip TS POC test in *T. solium* endemic communities.

### 2.2. Study Planning

The SOLID project had a multidisciplinary team with a very intense North/South collaboration. The project was funded for a period of four years. The entire first year served to elaborate on the study protocols, prepare the SOPs and treatment protocols, electronic case report forms (eCRFs), train project staff, and obtain the necessary ethical clearances and approvals. The trial was registered on 21 November 2017, at the Pan African Clinical Trials Registry (identifier: PACTR201712002788898). The SOLID study project was granted ethical clearance for the Zambian part of the study by the University of Zambia Biomedical Research Ethics Committee [UNZABREC 005-07-17], the Institute of Tropical Medicine [IRB/AB/ac/112 Ref 1177/17] through the ethics committee of the University of Antwerp [EC UZA 17/31/352], and by TUM through the Ethical committee of Klinikum rechts der Isar, Munich [299/18S]. Authorization to conduct research was also granted by the National Health Research Authority at the Ministry of Health in Zambia.

Participant recruitment was done in year two and three. Enrolment started in December 2017 and the last patient was recruited in July 2019. The fourth and final year was used for data cleaning, analyses, and result dissemination and communication to a large span of stakeholders ranging from study participants to ministries in Zambia and international organizations such as the WHO.

### 2.3. Study Objectives and Outcomes

The primary objective of the study was to assess the sensitivity and specificity of the TS POC T and TS POC CC strips for T and CC/NCC, respectively (Table 1).

Since there is no gold standard test for the detection of T nor of CC, three different reference tests were performed (Table 1), and a Bayesian approach was used to determine the accuracy of the TS POC test for T and CC [11]. For NCC, a composite case definition was used as reference standard according to predefined criteria [12].

A secondary outcome of the study was to assess the accuracy measures of the TS POC T test relative to results after the treatment and purging of participants who tested positive using the TS POC T and/or any of the T reference tests. The exploratory outcomes of the study included the assessment of observer agreement and ease of use of the test. The follow-up of patients diagnosed with active NCC (over a period of six months), the effect of anthelmintic treatment of symptomatic patients with active NCC, and the community prevalence and clinical/radiological characteristics of NCC were also evaluated.

Laboratory personnel performing the analyses of the reference tests for T and CC were blinded to the TS POC test result. CT scans were read in detail by two independent readers (blinded to the results of the other reader, clinical results, and the TS POC result). Details regarding the assessment of the primary outcome measures can be found in Appendix A.1.

### 2.4. Study Design

**Figure 1:**
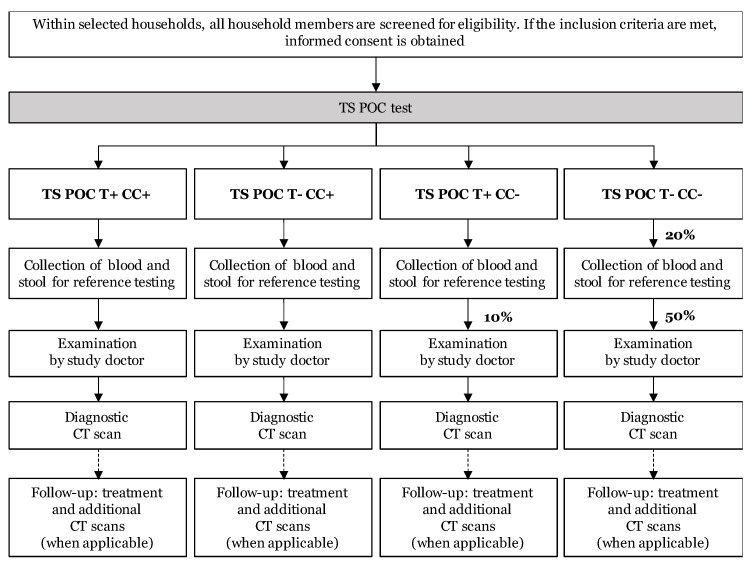
Flow of the community-based diagnostic accuracy study in Zambia within the SOLID project. In this two-stage design, all participants were tested using the TS POC test (=index test; stage 1), after which all positive and a subset of negative participants (indicated as percentages) were selected for reference tests, clinical examination, and a brain CT scan (stage 2).

The study was a diagnostic accuracy study with prospective data collection, using a two-stage design. All tests were applied to the same study subject (within-subject or paired design). The flow of the study is visualized in Figure 1. After obtaining written informed consent, all participants were subjected to the TS POC test (index test; stage 1). All participants with a TS POC positive result and a subset of participants with a TS POC negative result were subjected to several reference tests, clinical examination, and computed tomography (CT) scanning of the brain (stage 2).

The TS POC test was interpreted by two independent readers (two-factorial design) and provided results for both T and CC, resulting in four different TS POC test result combinations: T+CC+, T-CC+, T+CC-, and T-CC-. A blood sample was collected, and a stool sample was requested from all participants who tested positive for at least one test and a 20% subset of participants who tested negative for both outcomes. The 20% subset was obtained by selecting every 5th T-CC- participant for sampling, using a tally system.

All TS POC CC positive and a subset of the TS POC CC negative study subjects were invited for further (clinical) examinations (Figure 1). In the T+CC- group, a tally system was used, and every 10th participant was selected. From the T-CC- group that was selected for reference sampling, half was invited for further examination by tossing a coin, resulting in a final 10% of the T-CC- group that was selected for further (clinical) examinations. After a clinical neurological examination and a detailed questionnaire at the rural health center by a study doctor, the participants were invited for a diagnostic CT scan of the brain.

All participants who tested positive using the TS POC T test and TS POC T negative participants who tested positive using any of the reference tests for T, were treated with niclosamide and purged. For symptomatic patients with active NCC (based on the diagnostic CT scan), additional CT scans were performed at later time points to evaluate the treatment effect. More information regarding the treatment and follow-up of patients is given in Appendix A.2.

### 2.5. Study Area and Population

**Figure 2:**
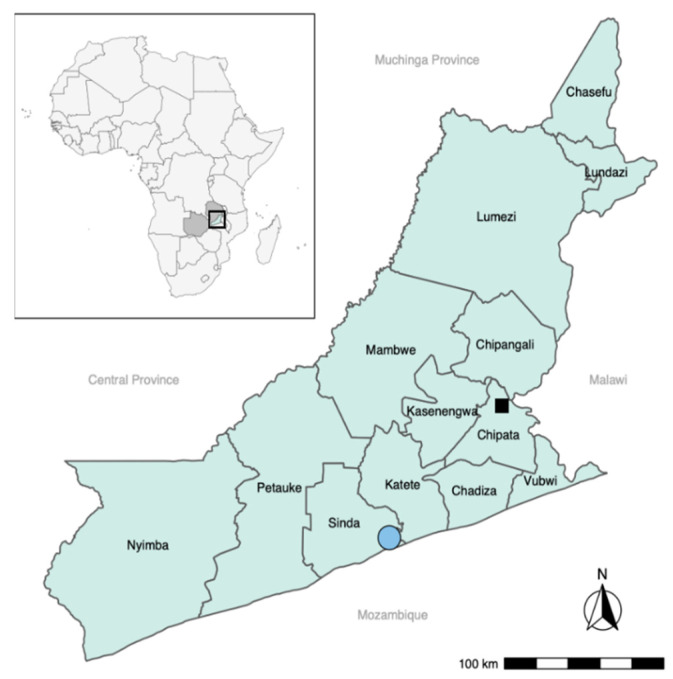
Map showing the study area in Eastern province of Zambia. The study area was the catchment area of Mtandaza Rural Health Centre in Sinda District (blue circle). CT scans were performed in Chipata Central Hospital in Chipata District (black square).

The study area was Sinda district in the Eastern province of Zambia (Figure 2). The area was selected as it was well known by the research team and recognized for its high *T. solium* endemicity, the presence of free-roaming pigs and low sanitation levels [13]. The prevalence in the Eastern province of Zambia of human cysticercosis is reported to be 33.5–38.5% (based on antibody detection), and of taeniosis of 6.3–11.9% (copro antigen ELISA) [4].

Communities were selected based on their willingness to participate, proximity to a Rural Health Centre (RHC), willingness of the staff to cooperate, and year-round accessibility. Four Neighborhood Health Communities (NHC; Mtore, Butao, Chinzure, and Ndaula) in the catchment of Mtandaza RHC were selected. These four communities comprised of 40 different villages in total.

### 2.6. Study Participants: Eligibility and Recruitment

The exclusion criteria were minimized as much as possible to obtain a representative sample of the target population, but certain groups were excluded due to ethical reasons (particularly related to the CT scan). The inclusion criteria were living in the area and being 10 years of age or older, while the exclusion criteria were pregnancy, and suffering from self-reported severe health conditions hampering daily activities.

Based on an informal pre-study census, the total population within the participating communities was 4331 inhabitants, belonging to 862 households. To obtain the targeted sample size of 1200 participants, 27.7% of our target population had to be tested. The number of participants in each village was thus determined proportionately (27.7%) to the total number of inhabitants within that village. 

All households within a village were randomized by generating a random number. Starting with the household with the lowest random number, all household members within the selected household were screened for eligibility, and every consenting eligible household member was recruited. Each participant was given a unique study code at enrolment. The number of household members who were not available and who were not willing to participate, were also documented. The recruitment of the next households continued until the target number for the village was met.

Registration (including written informed consent/assent), the TS POC test, and blood and stool sampling was conducted through door-to-door visits to the selected households by a qualified and trained researcher and (community) health worker. Written informed consent (assent and parent/guardian informed consent for minors) to take part in the study was sought from each participating individual prior to commencing the study procedures. For illiterate participants, a thumbprint was used, and a witness was consulted to sign on their behalf. An additional consent provision was included to store samples after completion of the study and future scientific research.

The study communities were sensitized, starting with the area Chief and village headmen to obtain permission to recruit people in their villages. Sensitizations for all villages in each of the four NHCs were held to explain the study. The sensitization was repeated a day before recruitment in the village commences, and comprised the explanation of the life cycle of the parasite, the purpose and methods of the study, and potential harms and benefits. This sensitization was repeated at the household level during recruitment.

### 2.7. Training

To ensure high quality data collection, everyone involved in participant recruitment and performing the TS POC test was trained on the biology of the parasite, diagnosis, treatment, prevention, and control of the parasite in endemic areas. Training on project procedures, Good Clinical (Laboratory) Practices and sample collection for nurses and community health workers and refresher trainings of all field teams were set up prior to each field mission to assure the proper handling and troubleshooting of the TS POC test prior to each sampling time point. The compliance on good storage and distribution practices along the supply chain was also documented, following a detailed Standard Operating Procedure (SOP) regarding TS POC test receipt, handling, storage, distribution, and retrieval of unused tests prior to implementation. GZ, who performed the clinical examinations and administered the questionnaires, was trained in neurological examination prior to the study and was supported throughout the study by the neurology team at TUM (ASW, DS) and ITM (EB).

### 2.8. Data Management

Specific eCRFs were created using EpiCollect5 (https://five.epicollect.net, accessed on 21 June 2021) to capture the participants’ data. Laboratory test results were recorded in Microsoft Excel files, using a separate database per reference test. Databases with results of the TS POC test, laboratory analyses, clinical examinations, and CT scans were cleaned separately, so personnel involved in data cleaning were blinded to the other test results. Databases were only merged prior to data analysis, after a sufficient level of cleaning of each of the databases. More information on data validation, monitoring, and adverse events can be found in Appendix A.3.

### 2.9. Sample Size and Statistical Analysis

The sample size of 1200 study subjects was calculated to obtain a desired precision of the sensitivity and specificity of minimum 10% [14,15].

The primary analysis question of this study was to determine the sensitivity and specificity of the TS POC test for the detection of, respectively T, CC, and NCC, each using (a set of) different reference tests. Due to the lack of a gold standard for T and CC, the diagnostic sensitivity and specificity of the TS POC test was estimated using a Bayesian approach [11]. The sampling scheme was accounted for in the analysis by expanding the original approach by altering the multinomial probabilities according to the observed sampling frequencies. For the diagnosis of NCC, the sensitivity and specificity were computed using the final NCC diagnosis as gold standard, also correcting for verification bias. More details on sample size calculation and statistical analyses can be found in Appendix A.4.

## 3. Discussion

The study was designed to minimize potential sources of bias and maximize generalizability, while accounting for the challenges of working in resource-poor community settings and limitations related to the disease, such as the low prevalence of taeniosis and the lack of gold standard for taeniosis and cysticercosis.

To increase the efficiency of the study, a two-phase design was used. All participants received the index test (TS POC), after which all TS POC positive participants and a sample of the TS POC negative participants were selected for reference tests. Consequently, the total number of reference tests, clinical examinations, and CT scans was reduced compared to a conventional cohort design. When the prevalence is low and the reference standard is expensive or invasive, a two-phase design is the design of choice [16]. The number of various reference tests, which were more time consuming and expensive than the index test, could thus be lowered. This reduction was particularly worthwhile for CT scanning, as it is expensive, logistically difficult to organize, and the inclusion of a disproportionate number of (unnecessary) brain CT scans in healthy participants would be unethical. Besides the benefits of increased efficiency, a drawback of a two-stage design is the risk of bias if there is a differential dropout, which is a potential problem in any study. Nevertheless, withdrawals can be higher in this type of designs, particularly if the reference tests are more time consuming or invasive than the index test and due to the potentially longer time period between performing the index test and reference tests [16]. The latter was avoided by requesting samples immediately after the TS POC test, which was feasible since the results of the TS POC test are available within 20 min [17], thus reducing loss to follow-up due to a time gap. 

The study initially envisioned more complex flows for sampling and clinical follow-up. As an example, for TS POC T-CC+ participants, only blood samples are needed as only serum is necessary for the CC reference tests. An additional stool sample for T reference testing would only be requested for a negative subset in this group. Nevertheless, a more uniform and simpler flow was finally adopted by requesting both blood and stool samples from all selected participants to avoid errors of incorrect sampling. Additional subsets were also planned for CT scanning, based on the combination of the TS POC test result and clinical examination results. Nevertheless, complex constructs are more difficult to implement under field settings, not only because of difficulties in identification, but also due to the lack of continuous internet access and technical resources, which hampers instant simple randomization and a complex follow-up system. The tally system that was finally used in this study to select subsets of participants for sampling, allowed immediate action, thus providing more clarity for the participants about the following steps in the study and avoiding a discontinuity between testing and sampling. Additionally, the simplified flow for clinical follow-ups also allowed participants to be informed without delay, which was preferred over an off-site randomization of participants. The latter would have resulted in a time lag and difficulties in seeking out selected participants, which may have resulted in more loss to follow-up. Several other measures were also taken to minimize attrition bias for the evaluation of NCC, such as regular visits to selected participants, organizing free transport to Chipata Central Hospital and providing incentives (food and drinks during CT scan appointments), and medical follow-up/treatment free of charge.

The project aimed to determine the accuracy measures of the TS POC test in communities that are highly endemic for *T. solium*. Therefore, all eligible household members within randomly selected households were recruited, to get a good representation of people with and without the disease in the target population. Nevertheless, pregnant women and children below the age of 10 years were not included for ethical and practical reasons such as the risk of radiation in case a CT scan would be needed, and the difficulties associated with sample collection, CT scanning and purging of young children. Although NCC is often associated with late onset epilepsy, the impact of NCC in children is not extensively studied, resulting in a research gap that needs urgent attention. Thus, future project proposals are encouraged to include also children. Also, people who were absent at the time of sampling or refused participation were not included in the trial, potentially leading to a bias in the results and limiting generalizability of the study results.

To avoid review bias, laboratory analyses were performed by personnel that was blinded to the TS POC results (and other reference test results). Reading of the CT scans was done by two independent readers, who were also blinded to the TS POC result. A two-factorial design was used, during which the TS POC was interpreted by two readers. While the training was necessary to ensure high quality data collection and correct recruitment, challenges for independent reading of the TS POC test appeared. Given the rural and door-to-door setting, it was difficult to ensure complete blindness by the second reader, which might lead to a bias in the results. Solutions, such as a window cover, were implemented. 

This community-based study was expected to define the accuracy measures for the TS POC test for the detection of T, CC, and NCC, for its use in the community. Besides the aforementioned benefits of increased efficiency, the two-phase design was thus also particularly useful as the index test was evaluated at a point in the diagnostic pathway where it is meant to be used in practice [16]. If the TS POC test for T proves to have a good sensitivity, the test can be used to screen people in the community. The early detection of tapeworm carriers is crucial to stop disease transmission as it ensures a rapid halt to environmental contamination with infective eggs, reducing not only the risk of new (N)CC cases, but also of porcine cysticercosis, and thus breaking the life cycle. A rapid test with high sensitivity is thus particularly useful for control programs against *T. solium*, allowing selective chemotherapy of people who test T positive during screening. Besides its usefulness in control programs, the simultaneous detection of T and CC also makes the TS POC test a valuable tool for epidemiological research, and to monitor the effectiveness of interventions of control or elimination programs [18].

The study also evaluated if the TS POC test has good predictive values for the detection of NCC, symptomatic or not, which could contribute to an improved diagnosis and referral of test positive people to district hospitals, where further diagnosis and follow-up can be made. Additionally, in areas with ongoing mass drug administration programs with praziquantel and/or albendazole, the test could potentially identify people that may be at risk for the presence of viable cysticerci in the central nervous system. Nevertheless, before the implementation of the tool as a screening device for NCC cases, additional studies must be performed to evaluate the cost-effectiveness of such strategies and whether screening with the TS POC test is also associated with improved patient outcomes [19]. An assessment of the clinical importance of the TS POC test should then preferably be done using a diagnostic randomized controlled trial [20].

## 4. Conclusions

This trial provides a rigorous evaluation of the TS POC test for the rapid and simultaneous detection of T, CC, and NCC in people living in communities highly endemic for *T. solium*. In case the accuracy is less than what was anticipated, the study will be able to provide information on how the TS POC test could be improved. If the proposed test is successfully validated, it can be proposed for implementation under specific settings. It may lay the groundwork for the improved control, monitoring, and surveillance of the disease by providing a tool for the rapid detection of tapeworm carriers and people who were exposed to tapeworm eggs. After a thorough assessment of the clinical importance, it may also enable a more rapid detection and referral of potential (asymptomatic) NCC cases to health centers and district hospitals, which would aid in improving the follow-up and health status in resource-limited communities. Moreover, the capacity and knowledge building component, including digitalization, which is inherently linked to this type of clinical trial, brings a lot of added value to the communities, by increasing awareness of the disease through sensitization meetings and training of health staff in health centers regarding diagnosis and patient management of tapeworm carriers and people with epilepsy.

## Figures and Tables

**Table 1:** Reference tests to assess the sensitivity and specificity of the TS POC test for different disease presentations of *T. solium*.

Disease Presentation	TS POC Strip (Index Test)	Reference Test	Target of the Reference Test
Taeniosis	TS POC T	Copro antigen ELISA	Antigens (in stool)
		Multiplex PCR	DNA (in stool)
		rES33 immunoblot	Antibodies (in serum)
Cysticercosis	TS POC CC	LLGP-EITB	Antibodies (in serum)
		rT24H immunoblot	Antibodies (in serum)
		Serum antigen ELISA	Antigens (in serum)
Neurocysticercosis	TS POC CC	Final diagnosis assigned by an expert panel, according to guidelines of Del Brutto et al. [12]	Cerebral CT scan, clinical presentation, epidemiological setting and serological results

TS POC T: *Taenia solium* point-of-care test, taeniosis test strip; ELISA: enzyme-linked immunosorbent assay; PCR: Polymerase Chain Reaction; TS POC CC: *Taenia solium* point-of-care test, cysticercosis strip; LLGP: lentil lectin-bound glycoproteins; EITB: enzyme-linked immunoelectrotransfer blot; CT: computed tomography.

## Data Availability

No new data were created or analyzed in this manuscript. Data sharing is not applicable to this article.

## Appendix A

### Appendix A.1. Primary Outcome Measures

#### Appendix A.1.1. TS POC Test

The TS POC is an antibody-detecting prototype test developed by a team of researchers from the CDC in collaboration with TUM and is based on two previously characterized and extensively used recombinant proteins, rES33 and rT24H [9,10]. This test is an in-house produced standard lateral flow assay (LFA), which consists of a double-strip cassette that holds two separate strips: one strip for the detection of taeniosis antibodies (by using the rES33 protein) and one for cysticercosis antibodies (by using the rT24H protein). Each LFA strip has one test line and one control line and a separate port for the sample application.

TS POC cassettes were sealed separately in an aluminium pouch together with a desiccant. Each complete TS POC kit was labelled with a batch number, production, and expiry date. For this study, all TS POC kits and chase buffer were shipped at room temperature from the CDC Atlanta to the University of Zambia (UNZA) and stored between 10 and 30 °C (TS POC tests) and 4–10 °C (chase buffer) until further transportation to the study area and use.

For the TS POC test evaluation in Zambia 2400 TS POC tests were produced at CDC Atlanta in collaboration with TUM and Arista Biologicals Inc, Allentown PA, USA. TS POC tests were produced in four batches during the project period. Details concerning reagents and procedures of each batch were documented according to good laboratory manufacturing practices. For quality assurance, each batch was pre-tested at CDC Atlanta with a defined positive and negative serum panel before shipment to Zambia (UNZA, School of Veterinary Medicine). The preliminary laboratory performance of the TS POC was estimated at a sensitivity of 88–93% (for one cyst or multiple cysts, respectively) and specificity of 99% for cysticercosis, and a sensitivity of 82% and specificity of 99% for taeniosis (CDC, TUM, 2017 unpublished).

Detailed information on how the TS POC test was performed and read can be found in Mubanga et al. [17]. In short, after disinfection, a fingertip was pricked with a lancet, 20 µL of whole blood was collected using a micropipette and placed in the sample well for TS POC T. The procedure was repeated for TS POC CC using a different micropipette. After applying two drops of chase buffer, the results were read 20 min after the start of the flow by two readers, and a third reader was included when the first two readers disagreed on the result. A test was considered positive when the test line was visible. In the event of an invalid result (e.g., control line missing), testing was repeated using a new TS POC cassette. The results were recorded on a result card and entered electronically in electronic case report forms (eCRFs).

#### Appendix A.1.2. Reference Sample Collection, Processing and Analysis

Stool samples were collected by giving participants stool pots. Stool was aliquoted into two 15-mL tubes. One aliquot was put in 10% formalin for copro antigen ELISA, the other in 70% ethanol for multiplex PCR. The aliquots were stored at room temperature until testing.

Blood was sampled by a trained nurse or clinician. Venous blood (3 mL) was collected by venepuncture from the arm into plain tubes, which were stored at 4 °C overnight. The sample was then centrifuged at 3000 rpm and serum was aliquoted in two cryovials, which were stored at −20 °C until reference testing. Serum samples were tested using EITB and antigen ELISA.

The copro antigen ELISA was performed in the Regional Reference Laboratory at UNZA in Lusaka, Zambia. All other tests were performed at the Institute of Tropical Medicine, Antwerp, Belgium. Samples were transported to the testing facilities following the required conditions. Laboratory personnel performing the analyses were blinded to the TS POC test result.

##### Copro Antigen ELISA

The copro antigen ELISA procedure was performed as reported previously by Allan et al. [21] and modified by Mwape et al. [5]. Briefly, the stool sample in formalin was mixed with an equal amount of phosphate buffered saline (PBS, Basingstoke, Hampshire, United Kingdom) and stands for 1 h with intermediate shaking. After centrifuging at 2000× *g* for 30 min, the supernatant was used in the ELISA. The 96 well polystyrene ELISA plates (Nunc^®^ Maxisorp, Roskilde, Denmark) were coated with capturing polyclonal antibody 2.5 µg/mL in carbonate-bicarbonate buffer (0.06 M, pH 9.6; Sigma-Aldrich, St Louis, MO, USA) and were incubated at 4 °C overnight. The plates were washed once with washing buffer (0.05% Tween 20 [Sigma-Aldrich, Saint-Quentin Fallavier, France] in PBS). The plates were blocked with 150 µL of 2% newborn calf serum (NBCS, Gibco, Auckland, New Zealand) in washing buffer and incubated for 1 h at 37 °C while shaking. After adding 100 µL of the sample, plates were incubated for 1 h while shaking. The plates were washed five times, after which they were incubated with 100 µL, 2.5 µg/mL dilution, biotinylated secondary polyclonal antibody (produced in-house, ITM, Antwerp, Belgium) in blocking buffer (PBS with 0.05% (*v*/*v*) Tween 20 and 2% (*v*/*v*) of NBCS) for 1 h at 37 °C with shaking. The plates were washed 5 times and incubated with 100 µL of 1:10,000 Streptavidin-horseradish peroxidase enzyme (Jackson Immunoresearch, Baltimore, MA, USA) in blocking buffer for 1 h at 37 °C with shaking. After 5 times washing, plates were incubated at room temperature with 100 µL of ortho-phenylenediamine (OPD, Thermo scientific, Rockford, IL, USA) in citrate buffer (1 tablet/10 mL, Sigma-Aldrich, St Louis, USA) and 10 µL hydrogen peroxide (Merck, Darmstadt, Germany) for 15 min in the dark. The reaction was stopped with 50 µL 4N sulphuric acid (Merck, Darmstadt, Germany) and the plates were read with a spectrophotometer at 490 nm and 655 nm. A sample was positive if the average OD of two wells was above 0.972.

##### Multiplex PCR

DNA extraction of stool samples (200 µL in 70% alcohol) was done using the QIAmp^®^ fast DNA stool mini kit (Qiagen, Hilden, Germany) according to the manufacturer’s instructions. DNA was stored at −20 °C till use. A multiplex PCR was performed according to Yamasaki et al. [22] with some modifications, to detect and differentiate *T. saginata*, *T. asiatica* and American/African and Asian genotypes of *T. solium* (*T. saginata* 827 bp, *T. solium* Africa/America 720 bp, *T. asiatica* 269 bp, and *T. solium* Asia 984 bp). The reaction was performed in a final reaction volume of 25 µL containing 5 µL DNA template, 2.5 µL of each primer (0.2 µM, Biolegio, Nijmegem, The Netherlands), 5 µL RNase-free water and 12.5 µL Multiplex PCR Master Mix (Qiagen, Hilden, Germany). The DNA fragments were amplified with the following optimized thermal cycling conditions: 95 °C/15 min followed by 40 cycles of 94 °C/30 s for denaturation, 58 °C/90 s for annealing, 72 °C/90 s for extension; and 72 °C/10 min for final extension. The PCR was done in a Biometra T3000 thermocycler (Westburg, Leusden, The Netherlands) and PCR products (5 µL) were visualized by electrophoresis in a 2% agarose gel at 100 v for 40 min, after which the gel was stained in ethidium bromide and visualized using UV light (Bio-Rad Gel Doc XR with Image Lab Software). The size of the amplified products was compared to a 100 base pair ladder. Only samples in which *T. solium* was detected, were considered positive.

##### LLGP-EITB, rES33 and rT24H EITB

The EITB was performed as described previously [23,24] with a few modifications. All strips were provided by CDC. For the recombinant strips, rES33 and rT24H lines for T and CC were combined in one strip. Briefly, 5 µL of serum sample was diluted in 500 µL of 0.3% tween in PBS and 5% non-fat dry milk on a tray. The mixture was rocked for 1 min, after which the EITB strips were added and incubated rocking overnight at 4 °C. The strips were washed 4 times with warm PBS-tween. They were then incubated with M-AH-IgG-PO (Jackson Immunoresearch, Baltimore, USA; 1/1000 in PBS-0.3% tween) for one hour. The strips were washed 3 times in PBS tween and 2 times in PBS only, after which they were incubated with 3,3′-Diaminobenzidine (DAB, 10 mg, Sigma Aldrich, St Louis, USA) and hydrogen peroxide substrate solution (Merck, Darmstadt, Germany) for 10 min. The developed strips were washed 10 times with de-ionized water to stop the reaction. The LLGP-EITB strip was considered positive if at least one band was visible.

##### Serum Antigen ELISA

The B60B158/B158 60 serum antigen ELISA was used as previously described [25]. Briefly, the samples were pre-treated by mixing 75 µL of each serum sample with an equal volume of freshly prepared 5% trichloroacetic acid (TCA, Sigma-Aldrich, Hamburg, Germany). After mixing, the samples were incubated for 20 min at room temperature, after which they were vortexed again and centrifugated for 9 min at 12,000× *g*. The supernatant (75 µL) was mixed with an equal volume of carbonate/bicarbonate buffer (0.156 M, pH 10; Na_2_CO_3_ and NaHCO_3_; Merck, Darmstadt, Germany), resulting in a final dilution of ¼ of the serum samples. Polystyrene 96-well ELISA plates (Nunc^®^ Maxisorp, Roskilde, Denmark) were coated with 100 µL monoclonal antibodies (5 µg/mL) in carbonate-bicarbonate buffer (0.05 M, pH 9.6; Sigma-Aldrich, St Louis, MO, USA) and incubated with shaking for 30 min at 37 °C, or overnight at 4 °C without shaking. The plates were washed once with washing buffer (0.05% Tween 20 in PBS), blocked with 150 µL of 1% NBCS and incubated shaking for 15 min at 37 °C. After discarding the blocking solution, 100 µL of pre-treated serum was added in duplicate and plates were incubated for 15 min at 37 °C while shaking. A conjugate control, substrate control, two positive and eight negative serum samples were included for quality control and determining the cut off. The plates were washed five times, after which they were incubated with 100 µL biotinylated secondary monoclonal antibody (ITM, Antwerp, Belgium) in blocking buffer (1.25 µg/mL) for 15 min at 37 °C while shaking. Then, plates were washed five times and incubated with 100 µL of 1:10,000 streptavidin-horseradish peroxidase enzyme (Jackson Immunoresearch, Baltimore, MD, USA) in blocking buffer for 15 min at 37 °C while shaking. After five washing steps, plates were incubated with 100 µL of OPD (Thermo scientific, Rockford, FL, USA) in 0.05 M citrate buffer (Sigma-Aldrich, St Louis, MO, USA) and 10 µL hydrogen peroxide (Merck, Darmstadt, Germany) for 15 min at room temperature in the dark. The reaction was stopped with 50 µL 4N sulphuric acid (Merck, Darmstadt, Germany) and the plates were read with a spectrophotometer at 492 nm and 655 nm. The cut off was calculated based on the OD values of the eight negative serum samples using a variation of the students *t*-test [26]. A sample was positive if the ratio of the mean of the samples to the cut off was >1.

#### Appendix A.1.3. Clinical Examination and Neuroimaging

A subset of study participants (Figure 1) was selected for a clinical neurological examination and a detailed neurological questionnaire. Selected participants were referred to the primary health facility (rural health centre of the study area) for a clinical neurological examination and a detailed questionnaire by the study doctor. Study subjects’ personal and clinical data were recorded in eCRFs. After clinical evaluation, including current and past medical history focussing on epileptic seizures, a CT scan of the brain with and without contrast was performed if there were no contraindications and the participant consented. The CT scans were performed by trained radiological staff at site at the Chipata Central Hospital. If the time between the initial TS POC test result and the CT scan was longer than 2 months, a new TS POC test for CC was performed. The results were recorded to assess a potential disease progression bias but did not influence the flow of participants.

For every CT scan, details of NCC typical lesions were recorded, such as the number, location and stage of lesions (i.e., vesicular, colloidal vesicular, granular nodular, nodular calcified), as well as other pathologies. CT scans were read in detail by two independent readers (blinded to the results of the other reader, clinical results, and the TS POC result). For the reference standard of NCC, a composite case definition according to the criteria suggested by Del Brutto et al. [12] was used, defined by a specialist team, based on clinical symptoms, the detailed results of the diagnostic CT scan, detection of antibodies in serum and epidemiological considerations. The final NCC diagnoses was divided into definite and probable.

### Appendix A.2. Patient Treatment and Follow-Up

Participants who tested positive for taeniosis using the TS POC test or any of the reference tests, were treated with niclosamide (single oral dose, 2 g) and were purged two hours later with 30 g magnesium sulphate. They were given a dish in which to defecate and the stool was checked for the presence of proglottids the following day. The species of recovered proglottids was confirmed using PCR-RFLP on parasite DNA extracts according to Geysen et al. [27]. Stool samples collected after treatment were also subjected to copro antigen ELISA and multiplex PCR as described above (secondary outcome-. Household members of patients diagnosed with taeniosis receive a medical follow up. They were fully informed on potential symptoms/signs of NCC and the need to go to the nearest health facility if they would become symptomatic.

The interpretation of the CT scans to determine the treatment and follow-up of patients, was performed by the study medical doctors and senior project neurologists. Patients were informed about their neuroimaging results during a follow-up appointment by a study doctor. If indicated, treatment was offered, according to national guidelines and the approach suggested by Winkler [28]. Symptomatic cases with active NCC at brain CT were treated with anthelminthic medication (albendazole 15 mg/kg body weight/day in two separate doses for 10 days; maximum of 1200 mg/day) if clinical condition and radiological results allowed. Together with anthelmintic medication, corticosteroids were administered (dexamethasone 12 mg/kg/day in a single dose, but the dose was adjusted according to NCC presentation and the course of the neurological condition under treatment). Corticosteroid therapy started one day before the start of anthelmintic therapy. Patients with cysts in eloquent areas, i.e., specific brain areas whose damage could produce major focal neurological deficits or even death, or patients with a large number of cysts were not treated with anthelmintic medication. All patients with epileptic seizures, with both active and inactive NCC and regardless of whether they were treated with anthelmintics or not, received an optimization of the antiepileptic drug regimen. Patients with radiologically confirmed NCC who were asymptomatic at the time were referred back to the routine health-care system, with reference documents and individual counselling in case they became symptomatic at a later stage. Patients with symptoms were followed up (including subsequent CT scans) 6 weeks and 6 months after treatment termination to monitor treatment success. For patients who were not treated with anthelmintic medication, the 6-month follow-up time point was after the initial CT scan.

### Appendix A.3. Data Management

Specific eCRFs were created using EpiCollect5 (https://five.epicollect.net, accessed on 21 June 2021) to capture participants’ data: to record eligibility criteria and all TS POC related results (CRF2), clinical examination, basic neurological examination, and an in-depth neurological questionnaire (CRF3-CRF5), CT scan results (CRF6), patient follow-up (CRF7), and TS POC re-test results (CRF8). All eCRFs were exported from EpiCollect5 and stored as comma-separated values (csv) files. CRF1 was used to record the name, contact details, and study code of the participant and was stored locally only.

Each of the eCRFs included real-time data validation rules whenever possible. Additional data validations were performed for each of the databases by the data management team at Ghent University (UGent) and the Institute of Tropical Medicine (ITM), who were not involved in data collection. This was done using R-scripts and reports were rendered using R Markdown [29] shortly after each field visit. Pictures of the TS POC test outcome and the TS POC results card were taken for all participants and these were used to cross check the electronically entered data. Barcodes were used to scan the study codes for electronic data collection whenever possible, to avoid entry errors. Regular reports (at least 2-monthly) were created using R-scripts to verify if all the necessary eCRFs were entered and whether patients received treatment/follow-up.

All data were saved on three different places, with one storage on-site and the others off-site on secured network drives with managed access. A backup was made of all electronic data at least once a week during ongoing field studies. Access to electronic forms was restricted to a limited number of users and users had different levels of access according to their role. Full access to the EpiCollect5 projects was restricted to people involved in data management to minimize unwanted changes and data loss.

In the event of an adverse event (AE)/severe adverse event (SAE), a system for AE/SAE reporting was set up with information on whom to contact, when, and the actions to take depending on the severity of each single case. Results were reported to the External Advisory Board (three external experts who were independent from the sponsor), which also took the role of Data Monitoring Committee. An internal audit was performed by the study sponsor (ITM) while recruitment was ongoing.

### Appendix A.4. Statistical Considerations

#### Appendix A.4.1. Sample Size

The sample size was calculated separately for T and CC to obtain a desired precision of the sensitivity and specificity of minimum 10% [14,15]. Assuming a prevalence of 35%, a sensitivity of 88%, and a specificity of 99%, 116 participants were required for CC. Assuming a prevalence of 5.9% for taeniosis, a sensitivity of 82% and a specificity of 99%, 961 participants were required. As the TS POC test is a combined test for T and CC, the highest sample size should be used for both tests, which was 961 for T using the anticipated sensitivity and specificity of the tests. However, when calculating the sample size for different sensitivities of the TS POC test (ranging between 98% and 75%), the sample size varied between 128 and 1221 for T and between 22 and 206 for CC. The final sample size was increased to 1200 to adjust for the possibility that the sensitivity of the TS POC test would be lower than initially anticipated.

#### Appendix A.4.2. Summary Statistics and Statistical Analyses

Results were reported according to the Standards for Reporting of Diagnostic Accuracy Studies (STARD) guidelines [30]. The TS POC test results (T and CC) were summarized, and the numbers and percentages of each result were presented. All tests yield dichotomous results.

For T and CC, the diagnostic sensitivity and specificity of the TS POC test was estimated using a Bayesian approach [11] due to the lack of a gold standard. The sampling scheme was accounted for in the analysis by expanding the original approach by altering the multinomial probabilities according to the observed sampling frequencies. Prior information to build the models was obtained via expert opinions. Model performance was checked using deviance information criterion, Bayesian *p* value, and effective numbers of estimated parameters [11]. Analysis was done using Open BUGS software version 3.2.3 (www.openbugs.net, accessed on 21 June 2021) and was applied only for observations that have a valid result for the TS POC test strip and reference tests under evaluation (complete case analysis). For taeniosis, results after treatment/purging (secondary outcome) were not included in the primary analysis since only participants who tested positive for any of the tests were treated. Treatment/purging results were only reported descriptively. The comparative performance of the different (reference) tests was also evaluated by cross tabulating the different test results (exploratory outcome).

For the evaluation of the TS POC test performance for the diagnosis of NCC, diagnostic accuracy measures were computed using the final NCC diagnosis as gold standard (primary outcome). The results of clinical examinations, CT scans, follow-ups and treatment evaluation were reported descriptively (exploratory outcomes).
